# Surface Modification of Cellulose Nanocrystals with Lactone Monomers via Plasma-Induced Polymerization and Their Application in ABS Nanocomposites

**DOI:** 10.3390/polym13162699

**Published:** 2021-08-13

**Authors:** Ramón Díaz de León, Ediberto Guzmán, Ricardo López González, Alejandro Díaz Elizondo, Ilse Magaña, Guadalupe Neira, Adali Castañeda Facio, Luis Valencia

**Affiliations:** 1 Research Center for Applied Chemistry, Blvd. Enrique Reyna 140, San José de los Cerritos, Saltillo 25294, CH, Mexico; ediberto.guzman@ciqa.edu.mx (E.G.); ricardo.lopez@ciqa.edu.mx (R.L.G.); alejandro.diazelizondo@ciqa.edu.mx (A.D.E.); ilsma.rivera58@gmail.com (I.M.); guadalupe.neira@ciqa.edu.mx (G.N.); 2 Facultad de Ciencias Químicas, Universidad Autónoma de Coahuila, Boulevard V. Carranza S/N, República Oriente, Saltillo 25280, CH, Mexico; adali.castaneda@uadec.edu.mx; 3 Biofiber Tech Sweden AB, Birger Jarlsgatan 57 C, SE-11356 Stockholm, Sweden

**Keywords:** cellulose nanocrystals, surface modification, plasma-induced polymerization, ε-caprolactone, δ-decalactone

## Abstract

The growing concern for environmental problems has motivated the use of materials obtained from bio-based resources such as cellulose nanocrystals which have a promising application acting as fillers or reinforcements of polymeric materials. In this context, in this article, plasma-induced polymerization is proposed as a strategy to modify nanocrystals at different plasma power intensities using ε-caprolactone and δ-decalactone to improve their compatibility with polymeric matrices. The characterization was carried out using techniques such as FTIR, TGA, XRD, XPS, and AFM, with which a successful functionalization was demonstrated without altering the inherent properties of the nanocrystals. The preparation of ABS nanocomposites was carried out with the modified nanoparticles and the evaluation of the mechanical properties indicates an increase in Young’s modulus and yield stress under certain concentrations of modified cellulose nanocrystals.

## 1. Introduction

Nanocellulose (NC) is often considered the next generation of renewable reinforcement for the production of high-performance biocomposites. Besides the high mechanical performance of the resultant materials, this is because of NC’s renewability and natural origin. Additionally, NC is much less abrasive than its inorganic and mineral equivalents for processing machinery, less hazardous for production employees in case of inhalation, easy to incinerate, leads to final compounds with a lower specific gravity (compared to mineral equivalents), and allows to obtain interesting properties in terms of thermal and acoustic insulation [1]. Moreover, the combination of a highly reactive surface (which allows the ease of surface modification) and great processability into 2D (e.g., films)/3D-materials (e.g., foams), makes NC an excellent candidate for the development of materials [2,3,4,5]. Nanocellulose can be divided into two main categories: Cellulose nanocrystals (CNC) and cellulose nanofibrils (CNF). Cellulose nanocrystals (CNC) were produced for the first time by Rånby (1949) [6] using acid hydrolysis of cellulose fibers in aqueous suspensions. Employing this treatment, CNCs are extracted with sulfate groups on their surface [7]. Some advantageous characteristics of CNCs compared to other bio-reinforcement materials include a high specific strength and modulus [8]. Moreover, other advantages can be found for CNC-reinforced nanocomposites, for instance, the incorporation of CNC typically leads to higher barrier properties with respect to water vapor and oxygen, and an enhancement of some aspects of mechanical properties. The use of natural and renewable materials, such as CNCs, appears as a great option for the development of “partially or fully” bio-based nanocomposite systems. However, the preparation of CNC-reinforced nanocomposites requires a surface modification strategy to improve the compatibility between the nano-reinforcement and the polymer matrix. For this purpose, several modification techniques have been previously reported, such as esterification reactions for introducing functional groups to nanocrystals, [9] as well as polymer grafting through controlled polymerization pathways [10,11,12]. Plasma-induced modification has emerged as an environmentally friendly alternative, where no solvent is required and leaves no residue that pollutes the environment [13]. Plasma-induced polymerization consists of the excitation of gas or monomer in the vapor phase through an electrical discharge, which polymerizes and precipitates on a substrate, forming an ultrathin pinhole-free polymeric coating [14].

**Figure 1:**
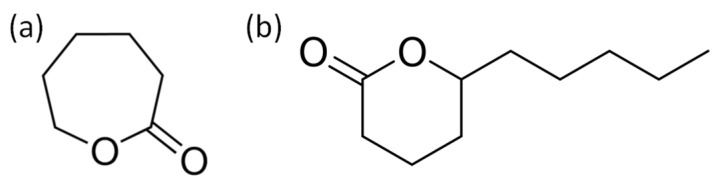
Chemical structure of (**a**) ε-caprolactone and (**b**) δ-decalactone.

The monomers’ ε-caprolactone and δ-decalactone (see Figure 1) belong to the group of lactones which are a class of esters that can be found in nature in the *Cryptocarya massoia* plant and castor oil [15,16]. These can be formed spontaneously by intramolecular reactions of hydroxy fatty acids and by oxidation reactions of their corresponding cyclic ketones [15]. They are widely used in the food industry and as precursors to produce bio-based and biodegradable polyesters such as polycaprolactone (PCL) [16]. Therefore, the study for its application in the surface modification of CNC represents a sustainable option. Additionally, polycaprolactone presents compatibility with other polymers such as PVC (polyvinyl chloride), poly (hydroxy ether), and SAN (styrene-acrylonitrile copolymer), [17] which suggests the potential for its use as reinforcement in ABS (acrylonitrile–butadiene–styrene) nanocomposites. ABS is a thermoplastic with different engineering applications such as car bodywork and protective equipment [18]. It being composed of two phases, a continuous one made up of SAN which imparts rigidity and brittleness to the polymeric matrix (amorphous), and the dispersed phase, which is made up of elastomer particles, generally polybutadiene, (PB) grafted with SAN, gives it the properties of elasticity and toughness, even under different temperature conditions [19]. In this work, the surface modification of cellulose nanocrystals via plasma-induced polymerization was studied using the monomers caprolactone and decalactone and applying different plasma input powers in the plasma reactor. To the best of our knowledge, there are no previous studies related to the use of decalactone under the conditions studied, and systematically studying different plasma powers to modify cellulose nanocrystals. Moreover, the preparation of ABS nanocomposites was carried out incorporating the modified nanocrystals.

## 2. Materials and Methods

### 2.1. Materials

Cellulose nanocrystals were purchased from CelluForce (Montreal, Canada). The ε-caprolactone and δ-decalactone monomers for the surface modification were provided by Sigma-Aldrich and used as received. Commercial ABS (ELIX ABS P2H-AT) with molecular weight (M_w_) of 119 kg/mol, dispersity (Ð) of 3, 21.6% of gel content, and swelling index of 3.8% was supplied by ELIX Polymers (Tarragona, Spain).

### 2.2. Plasma-Induced Polymerization

The surface modification via plasma-induced polymerization was carried out in a custom-made plasma reactor and considering the experimental procedures described by Neira et al. [20]. Our previous work shows a schematic representation of the equipment used [21]. In the reactor chamber, 1.5 g of CNC’s were introduced, and then the system was subjected to vacuum until reaching 1.1 × 10^−1^ mbar of base pressure. The monomer (ε-caprolactone and δ-decalactone) was introduced and allowed to flow until reaching a stable pressure of 1.3 × 10^−1^ mbar. Subsequently, the plasma source was induced with the radio frequency generator at different plasma powers (see Table 1). The CNCs were treated for 1 h at a stirring speed of 30 rpm. The resultant nanocrystals, after modification, are hereinafter referred to as modified cellulose nanocrystals (*m*-CNCs).

### 2.3. ABS Nanocomposites

The ABS/*m*-CNC nanocomposites were prepared in a Brabender mixing chamber using CAM-type rotors at a temperature of 200 °C and a speed of 60 rpm. In all cases, the 70 cm^3^ chamber was filled to 90% of its capacity. The ABS was first introduced into the mixing chamber until it was completely softened and then the *m*-CNCs were gradually added, leaving 5 min to mix. The nanocomposites were extracted from the chamber and later were compression molded in hydraulic presses using 80 × 80 × 3.2 mm plates. Compression molding was carried out at 210 °C with a pressure of 15 tons and cooled for 10 min. Type V specimens were prepared according to the specifications of the ASTM D-638 standard. Before carrying out the stress and impact resistance measurements, the specimens were conditioned at a temperature of 23 ± 2 °C and relative humidity of 50 ± 5% for 40 h. The formulations of the ABS nanocomposites are described in Table 2.

### 2.4. Characterization of Surface-Modified Cellulose Nanocrystals

The chemical composition of the CNCs was determined by Fourier Transform Infrared Spectroscopy (FTIR) using the Attenuated Total Reflection (ATR) technique, using a Thermo Scientific spectrophotometer model Nicolet iS50 (Thermofisher Scientific, Waltham, MA, USA). The samples were analyzed in a spectral region between 4000 and 400 cm^−1^ with a 4 cm^−1^ resolution and 32 scans.

The influence of the surface modification on the thermal degradation behavior of the modified CNC was studied by Thermogravimetric Analysis (TGA), using a TA 2000 thermo-analyzer (TA Instruments, New Castle, DE, USA). The analyses were carried out in a nitrogen atmosphere, with a gas flow of 20 mL/min from 25 to 600 °C at a heating rate of 10 °C/min.

The crystallinity of the *m*-CNCs was evaluated by X-ray diffraction (XRD), using an Ultima IV Rigaku equipment (Rigaku, Tokyo, Japan) under the following conditions: Cu Kα radiation (λ = 0.154059 nm), generator at 40 mA, and a voltage of 45 kV at room temperature. Angular exploration was performed at 5 to 60° at 5°/min. The size of the crystallite τ (nm), perpendicular to the lattice plane (002) of cellulose I, and the degree of crystallinity were calculated using the Scherrer [22] and Segal [23] equations, respectively.

X-ray Photoelectron Spectroscopy (XPS) to determine the surface chemical structure of the nanocrystals after modification was carried out with a Riber LDM-32 spectrophotometer (ISA-RIBER, Ruel-Malmaison, France) with an aluminum anode, using monochromatized A1 Kα radiation operating at 150 W with one step of 20 eV for the individual photoelectron lines. The high-resolution C 1s spectrum was fitted using a Shirley background subtraction and a series of Voigt peaks.

The morphology of the modified cellulose nanocrystals was observed by Atomic Force Microscopy (AFM) using a Nanoscope V system (VEECO Instruments, Santa Barbara, CA, USA). The CNCs were previously dispersed in deionized water and sonicated for 30 min; then, a drop of the diluted solution was deposited on a normal microscope slide and allowed to dry at room temperature. AFM images were tapping in the air at room temperature. From the obtained micrographics, mean length and diameter values were determined using ImageJ software (Fiji distribution, open-source, 1.8.0, National Institutes of Health, New York, NY, USA).

### 2.5. Characterization of ABS Nanocomposites

Tensile testing was carried out on a Universal Instron model SF-120 equipment (Instron, Norwood, MA, USA) following the ASTM D-638 standard. Moreover, Young’s modulus was obtained with the slope of the stress–strain curve. The test was carried out with a 5 kN cell, at a speed of 1 mm/min using type V specimens.

The impact testing of the prepared nanocomposites was determined by the IZOD test in a CSI model 137 equipment (Custom Scientific Instruments, Easton, PA, USA), following the ASTM D-256 standard.

The viscoelastic properties of the samples were measured in compression-molded samples in a Dynamic Mechanical Analyzer (TA Instruments, New Castle, DE, USA), in bending mode from 30 to 150 °C, using a frequency of 1 Hz and a heating rate of 10 °C/min.

The morphology present in the ABS was analyzed by TEM, using FEI-TITAN 80–300 kV equipment (ThermoFisher Scientific, Waltham, MA, USA). The samples were trapezoid sectioned from the specimens used for the impact test and then cut to a thickness ~ 90 nm with a Leica Ultracut UCT cryogenic ultramicrotome equipment with a diamond blade. The operating conditions were kept at a temperature of −125 °C. Additionally, the pure ABS sample was stained with osmium tetraoxide (OsO_4_) vapors for 2.5 h to contrast the phases; while the ABS composite samples were stained using phosphotungstic acid to achieve a contrast between the polymeric matrix and the reinforcing phase.

## 3. Results and Discussion

### 3.1. Surface Modification of CNC

**Figure 2:**
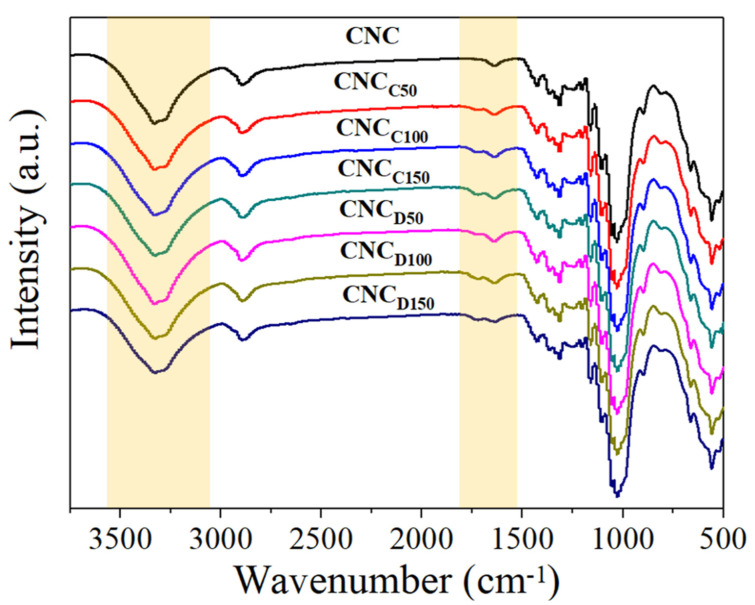
FTIR spectra of CNC modified with ε-caprolactone and δ-decalactone by plasma-induced polymerization.

The surface modification of the nanocrystals was demonstrated by FTIR spectroscopy, as shown in Figure 2. In general, the characteristic bands of cellulose are shown and did not show changes for the samples of pristine and *m*-CNCs (modified cellulose nanocrystals) [24]. This includes the peaks at 2898 and 1425 cm^−1^ corresponding to the stretch bands of the C–H groups and the flexion of the –CH_2_ groups. The band at 1050–1100 cm^−1^ was caused by C–O group vibrations, and that corresponding to 700–720 cm^−1^ was related to the oscillating vibrations of the C–H and H–C–H groups. In the FTIR spectra, the *m*-CNCs showed the appearance of a peak around 1700 cm^−1^ which displayed the contribution of the deposited polymers via plasma-induced polymerization, in this case of the ester (carbonyl contribution) groups of ε-caprolactone and δ-decalactone.

**Figure 3:**
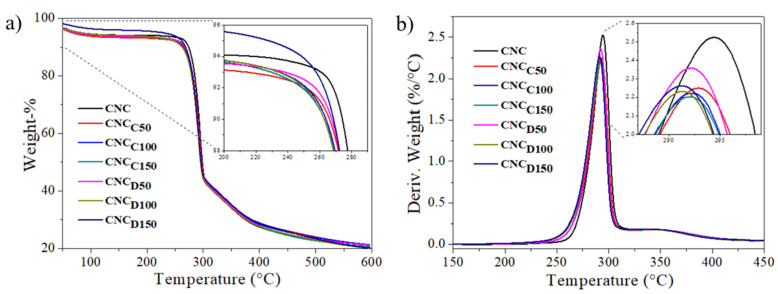
TGA thermogram (**a**) and mass loss rate (**b**) of CNCs and *m*-CNC’s.

The TGA thermograms of the cellulose nanocrystals modified by plasma with ε-caprolactone and δ-decalactone at the different powers are shown in Figure 3a. Weight losses were observed in three steps: the first weight loss that occurred below 125 °C can be attributed to the evaporation of adsorbed water; additionally, two degradation steps around 250 and 325 °C were evident, attributed to the first part of cellulose degradation, the beginning of the breakdown of glycosidic bonds; the third weight drop was attributed to the second part of cellulose decomposition, which was consistent with what has been reported in the literature [25]. Overall, the *m*-CNCs started to degrade slightly faster than pristine CNCs, which presumably happened due to the presence of low molecular weight polylactones (deposited upon plasma-induced polymerization), which degrade at lower temperatures than cellulose [26,27]. The small difference in the degradation profiles evidenced the low amount of deposited polymer via plasma-induced polymerization, as shown in previous works [28]. Nevertheless, despite the surface chemical differences that may exist in CNCs and *m*-CNCs, the thermal degradation behavior was rather consistent and ruled by the degree of polymerization of the chains of cellulose [25,28].

**Figure 4:**
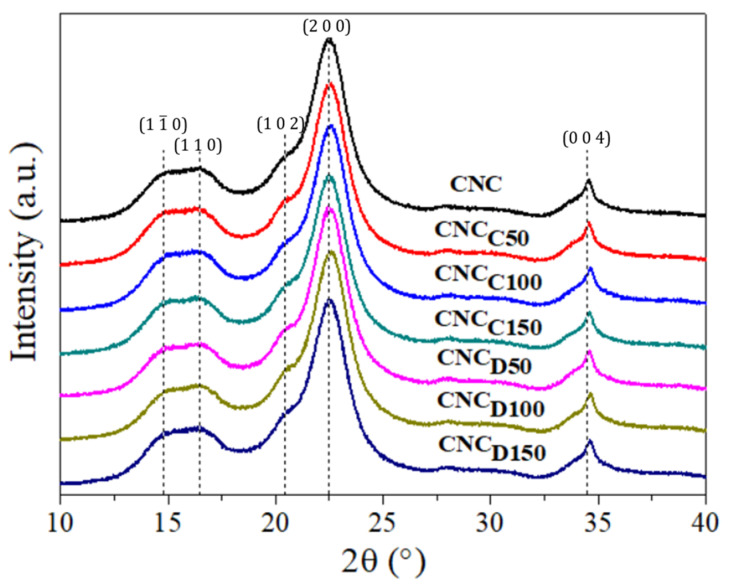
XRD diffractograms of CNCs and *m*-CNCs.

The crystallinity of the CNCs and *m*-CNCs was evaluated by X-ray diffraction (XRD) and the diffractograms are shown in Figure 4. The diffraction peaks located at an angle 2θ 14.95°, 16.4°, 20.6°, 22.5°, and 34.5°, which correspond to the crystallographic planes with Miller indices, can be indexed as (1 $\bar{1}$ 0), (1 1 0), (1 0 2), (2 0 0), and (0 0 4), respectively. The results indicate that the nanocrystals corresponded mainly to type I cellulose, with a high crystallinity index; this form of cellulose had the particularity that it showed a diffraction peak around 20.5°, which corresponds to crystals with Miller’s index (1 0 2) and this is particularly because this peak did not always appear in all type I cellulose samples [29]. Table 3 contains the calculated crystallite size (τ) values, having an average value around 4.0 nm (using the Scherrer equation), which is similar to the literature values reported for CNC derived from wood pulp [21,24]. In general, the same crystalline planes were observed for all the samples and a similar degree of crystallinity (X-%), being the highest for the pristine CNC, so it is suggested that the plasma treatment slightly increased the content of the amorphous phase, expectable due to the amorphous nature of the deposited lactone polymers onto *m*-CNCs via plasma-induced polymerization.

**Figure 5:**
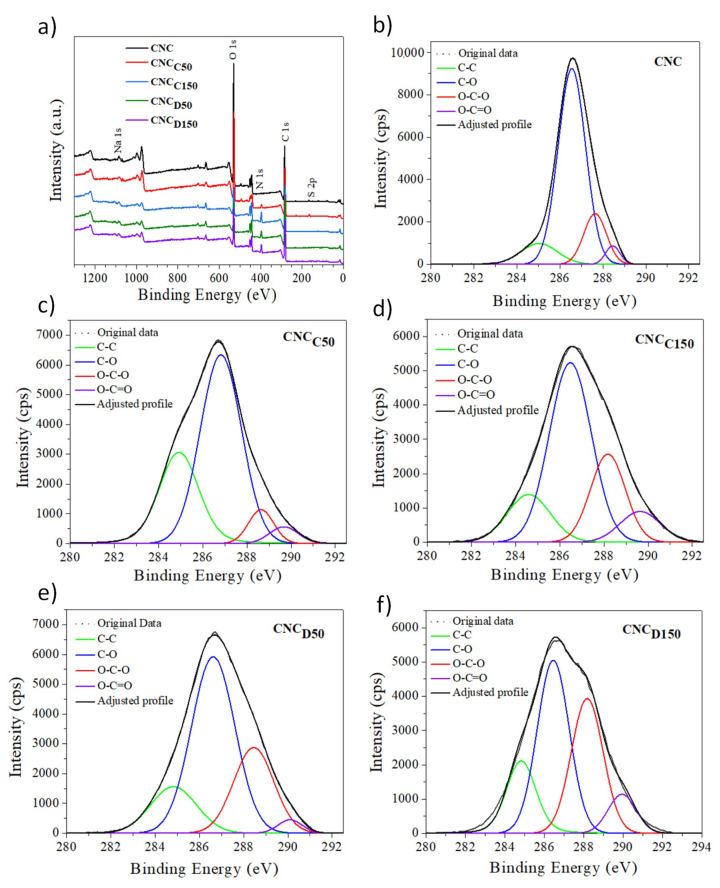
XPS survey (**a**), peak-fitted high-resolution of C 1s XPS spectra of the pristine (**b**) and modified CNCs (**c**–**f**).

The surface chemistry of cellulose nanocrystals was further studied, before and after modification, by XPS. Figure 5a shows the spectra obtained, where the main photoelectron lines for cellulose, C 1s, and O 1s, were observed. Additionally, a very small amount of sodium and sulfur (Na 1s and S 2p) was detected, arising from the sulfate semi-ester groups grafted on the surface of the CNC during its production by hydrolysis with sulfuric acid of wood pulp, and their associated sodium counterions. Figure 5b–f correspond to the deconvolution of the high-resolution C 1s spectra of the samples; according to the literature, this signal was decomposed into four populations. The first peak at 285 eV was assigned to the C–C environment [30]. The peak at 286.9 eV corresponds to an environment where carbon was attached to an oxygen C–O of which there are five atoms in the anhydroglucose unit. The component at 288.3 eV corresponds to a carbon bound to two oxygen O–C–O of which there is an atom in the repetitive structure of cellulose [31]. Furthermore, a fourth small population of around 289–290 eV was also observed, corresponding to the presence of carboxyl groups, present before and after the modification. The significant increase in the population corresponding to aliphatic carbon (C–C) according to Table 4 confirms the presence of the polymer deposited on the surface of the nanocrystals [31,32]. Furthermore, the signal corresponding to carboxyl groups and ester groups, which are present in pristine CNCs, increased after modification. This gives us an approximate notion of the amount of polymer on the surface of the nanocrystals. Likewise, the C/O ratio increased after the modification, associated with the aliphatic structure of the deposited polymer layer. Taking this as a reference, it can be established that the *m*-CNCs exposed to plasma of 150 W suffered a greater modification.

**Figure 6:**
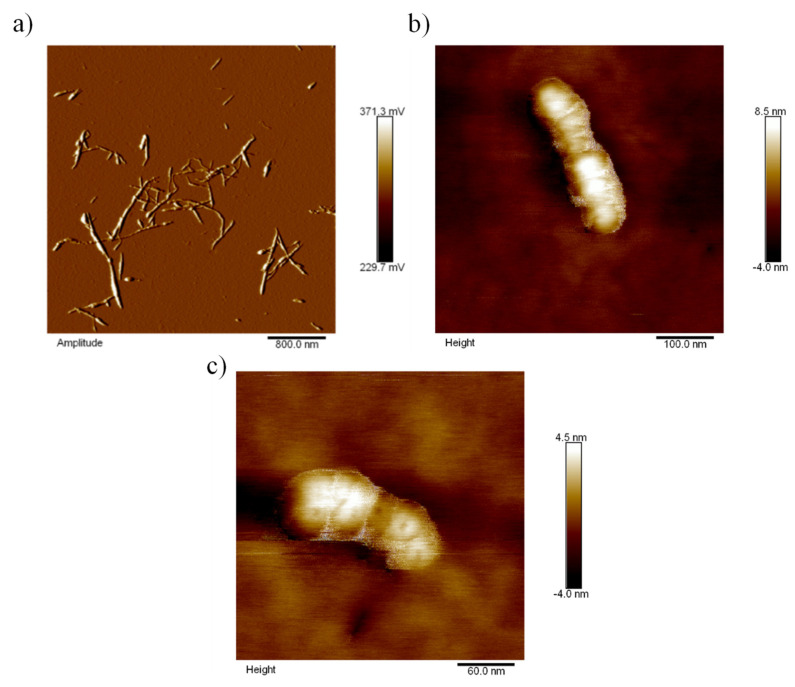
Micrographs obtained by AFM of pristine (**a**) and 150 W ε-caprolactone-modified cellulose nanocrystals (**b**,**c**). Micrographs were taken at different magnification to show the size of the neat nanoparticles and the influence of the surface treatment.

The morphology of the nanocrystals, before and after modification was, furthermore, studied via AFM. As it can be observed in Figure 6a, the pristine CNCs displayed nanoscale dimensions and a rod-like morphology with an aspect ratio of about 20. Figure 6b,c show the micrographs from the *m*-CNC after plasma treatment with ε-caprolactone for 1 h with a power of 150 W (CNC_C150_). In this case, it was observed that the resultant m-CNCs exhibited a rough surface with an evident layer of deposited polymer, which caused a decrease in the aspect ratio but preserved the rod-like shape.

### 3.2. ABS Nanocomposites

**Figure 7:**
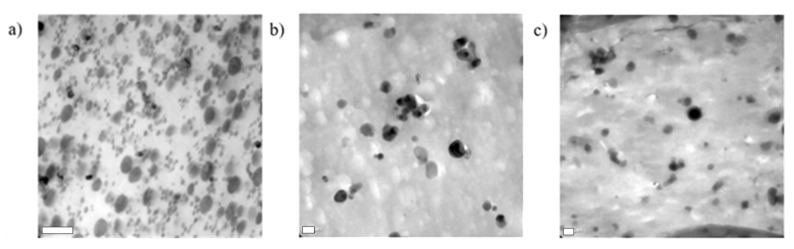
TEM micrographs of pure ABS (**a**), ABS/1.5CNC_C150_ (**b**), and ABS/1.5CNC_D150_ (**c**). Scale bar equal to 0.2 µm.

The incorporation of the *m*-CNSs into an ABS matrix was carried out, to elucidate whether the surface modification improved the compatibility of the nanofiller with polymer matrices. A pre-polymerized ABS was used, and the compounding was carried out through a Brabender mixer, followed by thermoforming. The *m*-CNCs obtained with a plasma power of 150 W for one hour were used (with both monomers) as this was the condition that generated the highest surface modification onto the nanocrystals. The morphology of the ABS materials was studied through TEM (see Figure 7a–c). Typical solid elastomeric particles associated with ABS emulsion can be observed, where the elastomer phase showed an average particle diameter (Dp) of 0.28 ± 0.11 µm [33]. By incorporating cellulose nanocrystals, one can observe that the presence of “white” areas was observed, which may correspond to the presence of agglomerates.

**Figure 8:**
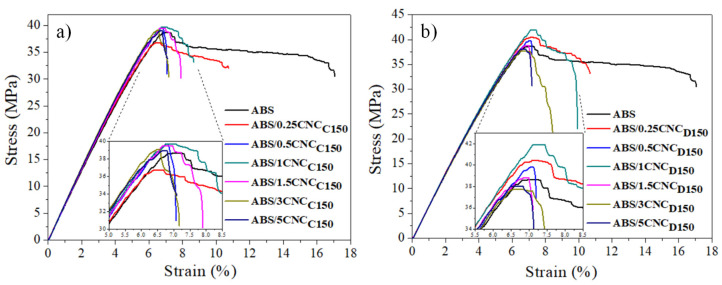
Stress versus strain curves of ABS/150W ε-caprolactone modified CNC nanocomposites (**a**) and ABS/150 W δ-decalactone-modified CNC nanocomposites (**b**).

The mechanical properties of the resultant ABS nanocomposites were studied via tensile testing, and the resultant stress–strain diagrams are shown in Figure 8a,b. In general, it can be observed that the behavior in the tensile test of all the nanocomposites was characteristic of a semi-rigid material, due to the contribution provided by the SAN matrix and the flexibility provided by the rubber particles dispersed in this matrix. For nanocomposites with *m*-CNCs modified with ε-caprolactone (CNC_C150_), a slight increase in the yield stress was observed by incorporating 1 and 1.5 wt% of nanocrystals. By increasing further, the yield stress values were lower than those obtained for the pure ABS. In contrast, in the case of *m*-CNCs modified with δ-decalactone, the resultant ABS show an increase in the yield stress with loadings of up to 1 wt%. This decrease in tensile properties presumably happened due to the optimum concentration being exceeded; presumably, a CNC saturation occurs due to aggregation. The improvement in this tensile property can be attributed to an effective stress transfer between the *m*-CNC and the ABS components responsible for the mechanical performance, that is, the SAN matrix and the elastomeric particles. The different behavior regarding the yield stress of the modified CNC with caprolactone and decalactone was attributed to the fact that the presence of the polymeric deposit promoted by δ-decalactone presumably contributed better towards the interfacial compatibility between the matrix and the nanofiller. It was also observed that the deformation or strain was deteriorated by the addition of CNC regardless of its modification, which was consistent with what is reported in the literature [34] when ABS is reinforced with rigid nanostructures; this, therefore, causes a reduction in the stress to the breakdown of the material.

**Figure 9:**
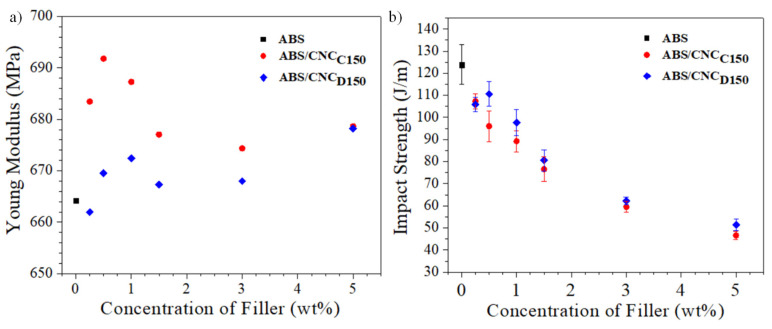
Young’s modulus (**a**) and impact strength (**b**) of ABS/*m*-CNCs nanocomposites.

Young’s Modulus values were determined from the slope of the stress–strain curves of the nanocomposites obtained (see Figure 9a). As it can be observed, there was an apparent trend regarding the addition of *m*-CNCs with both ε-caprolactone and δ-decalactone, where there was a maximum in Young’s modulus close to 1 wt% of *m*-CNC, and then this property fell and increased back at 5 wt%. This behavior may be associated with the concept of the percolation threshold in which, close to 1% by weight of the *m*-CNC concentration, there was intimate connectivity between the nanostructures such that it allowed promoting the increase in the property, in this case, the Young’s Modulus, and later, the property suffered a decrease due to a saturation of the *m*-CNCs. When comparing Young’s modulus values of the different ABS, those containing modified CNC with ε-caprolactone presented higher values. This may be associated with the fact that, since there was a lower affinity for ABS, aggregates of cellulose nanocrystals may have formed with the potential to increase Young’s modulus [35]. Finally, all nanocomposites showed a decrease in impact resistance as the concentration of *m*-CNC increased, as seen in Figure 9b; these results coincide with that reported in the literature, [36] which shows that the resulting materials increased their rigidity due to the addition of *m*-CNC, as a consequence of their high Young’s modulus [37].

**Figure 10:**
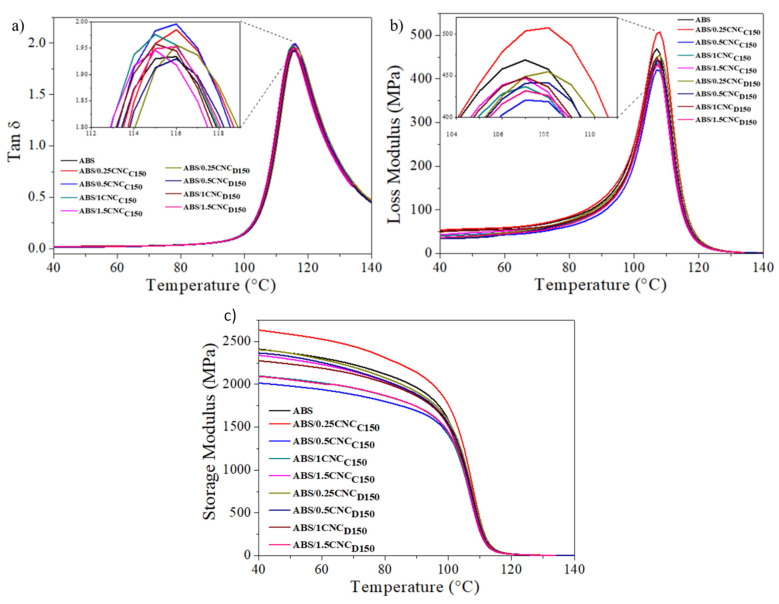
Tan δ (**a**), loss modulus (**b**), and storage modulus (**c**) of the ABS/modified CNC nanocomposites studied.

The influence of the nanocrystals over the glass transition temperature (T_g_) of the ABS nanocomposites was investigated from DMA. Figure 10a,b show the curves of the loss factor (Tan δ) and loss modulus against temperature, respectively. The peak in the curves can be associated with the transitions of the continuous phase (SAN) and the glass transition temperatures (T_g_) were determined from the peaks of the loss modulus and loss factor curves. The values are shown in Table 5. No further thermal transitions were observed due to the amorphous nature of ABS. Moreover, there was no drastic change in the glass transition temperature, remaining almost constant when adding the cellulose nanocrystals. By looking at the Tan δ (Figure 8a), which is related to the amount of energy dissipated by the material, one can observe that the nanocomposites, including *m*-CNCs, presented slightly higher values compared to pure ABS, which indicates that their incorporation into the polymeric matrix may reduce the elastic behavior of the material [21,38]. In Figure 10c, the storage module of the ABS nanocomposites is shown, in which it can be observed that the ABS/0.25CNC_C150_ nanocomposite presented the highest elastic modulus in relation to pure ABS. Young’s modulus values evaluated through stress tests were much lower than the storage modulus determined by DMA, possibly because the material was not completely isotropic and in addition to the fact that stress was not applied to the direction of the specimen [39].

## 4. Conclusions

This work corroborated the potential of surface-modifying cellulose nanocrystals via plasma-induced polymerization using ε-caprolactone and δ-decalactone as monomers, as suggested via FTIR and XRD, showing as increase the FTIR peak around 1700 cm^−1^ corresponding to the carbonyl groups of the deposited polyesters, and a slight decrease in crystallinity, as both polyesters are amorphous. The modification was further demonstrated via XPS mainly from the significant increase in C–C bonds (285 eV) and the C/O ratio which corresponds to the aliphatic nature of the polyesters. Moreover, different plasma potencies were used, displaying the influence over the modification degree which, according to the XPS analysis, the nanoparticles treated with plasma at a power of 150 W exhibited a greater surface modification using both lactone monomers: ε-caprolactone and δ-decalactone. The surface treatment did not alter the rod-like morphology of the nanocrystals, as shown via AFM.

ABS nanocomposites were prepared with some of the modified nanocrystals, and the results showed that the nanocomposite samples exhibited a typical ABS morphology with solid elastomeric particles dispersed within the SAN phase. The ABS samples were studied by mechanical tests and the results showed improvements in Young’s modulus and the yield strength compared to neat ABS, proving an optimum nanocrystal concentration of 1 wt% for both CNC and *m*-CNCs, but significantly higher values were shown when using the modified nanoparticles. The impact strength, however, was reduced as a function of nanocrystal content, due to the increase in stiffness of the biocomposite. No change in the glass transition temperature of ABS was shown upon CNC incorporation, and the highest storage modulus was shown by the composite with 0.25 wt% *m*-CNC, as determined via DMA. This work contributes to the employment of renewable resources towards the development of high-performance materials, utilizing environmentally friendly surface modification routes.

## Figures and Tables

**Table 1:** Monomers and plasma powers of plasma used in the surface modification by plasma-induced polymerization.

*m*-CNC	Monomer	Plasma Power (W)
CNC_C50_	ε-caprolactone	50
CNC_C100_	ε-caprolactone	100
CNC_C150_	ε-caprolactone	150
CNC_D50_	δ-decalactone	50
CNC_D100_	δ-decalactone	100
CNC_D150_	δ-decalactone	150

**Table 2:** Formulation of the ABS nanocomposites.

Sample	Filler	Concentration (wt%)
ABS	−	−
ABS/0.25CNC_C150_	CNC_C150_	0.25
ABS/0.5CNC_C150_	CNC_C150_	0.5
ABS/1CNC_C150_	CNC_C150_	1.0
ABS/1.5CNC_C150_	CNC_C150_	1.5
ABS/3CNC_C150_	CNC_C150_	3.0
ABS/5CNC_C150_	CNC_C150_	5.0
ABS/0.25CNC_D150_	CNC_D150_	0.25
ABS/0.5CNC_D150_	CNC_D150_	0.5
ABS/1CNC_D150_	CNC_D150_	1.0
ABS/1.5CNC_D150_	CNC_D150_	1.5
ABS/3CNC_D150_	CNC_D150_	3.0
ABS/5CNC_D150_	CNC_D150_	5.0

**Table 3:** Crystallographic parameters of CNCs and *m*-CNCs.

Sample	*I*^a^ (200), Cps	*Ia*^b^, Cps	χ (%)	τ ^c^, Nm (200)
CNC	14,949	1866	88	4.1
CNC_C50_	15,034	2038	86	4.1
CNC_C100_	15,178	2252	85	4.0
CNC_C150_	14,321	1908	87	4.1
CNC_D50_	15,428	1998	87	4.1
CNC_D100_	15,065	1733	87	4.1
CNC_D150_	15,072	2371	84	3.9

^a^ Total intensity of the (200) peak for cellulose I. ^b^ Intensity at 18° which is considered as the dispersion caused by the amorphous phase. ^c^ Crystallite size calculated from X-ray diffractogram.

**Table 4:** Surface composition of pristine and modified CNCs calculated by XPS and the C 1s high-resolution data.

Sample		Binding Energy (eV)			
	C/O	285 (C–C)	287 (C–O)	288 (O–C–O)	289 (O–C=O)
CNC	1.39	10.14%	71.18%	15.14%	3.54%
CNC_C50_	1.89	29.91%	59.80%	6.56%	3.72%
CNC_C150_	5.20	14.91%	54.18%	21.99%	8.91%
CNC_D50_	5.33	15.85%	56.50%	25.28%	2.37%
CNC_D150_	3.67	17.41%	41.92%	32.42%	8.25%

**Table 5:** Glass transition temperatures (T_g_) of the ABS/modified CNC nanocomposites obtained by DMA.

Sample	T_g_ (°C) Loss Modulus	T_g_ (°C) Tan δ
ABS	107.1	115.5
ABS/0.25CNC_C150_	107.6	116.0
ABS/0.5CNC_C150_	107.5	115.6
ABS/1CNC_C150_	106.9	115.0
ABS/1.5CNC_C150_	105.0	113.0
ABS/0.25CNC_D150_	107.8	116.0
ABS/0.5CNC_D150_	107.5	116.0
ABS/1CNC_D150_	107.0	115.5
ABS/1.5CNC_D150_	107.3	115.5
